# Editorial bullying: an exploration of acts impacting publication ethics and related environment

**DOI:** 10.3389/frma.2024.1345553

**Published:** 2024-02-21

**Authors:** Fawad Javed, Dimitrios Michelogiannakis, P. Emile Rossouw

**Affiliations:** Department of Orthodontics and Dentofacial Orthopedics, Eastman Institute for Oral Health, University of Rochester, Rochester, NY, United States

**Keywords:** academic bullying, editor, ethics, harassment, scientific publishing, peer-review, personal criticism, intimidation

## Abstract

Bullying and misconduct in the realm of scientific and scholarly publishing have the potential to jeopardize the transparency and integrity of academic discourse. While misconduct issues among authors have been extensively discussed, the role of editors in perpetuating or mitigating such problems has garnered less attention. Scientific publishing serves as the gateway for disseminating innovative research findings globally, and the role of editors, especially Editor/s-in-chief, is pivotal in safeguarding the rigor and credibility of published research. Editor bullying and misconduct involve behaviors that undermine the scientific process, compromise research integrity, and harm the careers and wellbeing of individuals. These actions may manifest as biased decision-making, suppression of dissenting voices, or the exploitation of power dynamics in the peer review process. To address these issues, preventive and therapeutic approaches are suggested, including enhancing awareness, recognizing and mitigating exacerbating factors, and upholding professionalism. Moreover, the importance of a conflict-of-interest declaration for editors is highlighted to ensure transparency and integrity in the editorial process. The present mini-review aims to shed light on editor bullying, illuminating its gravity and the urgency to address these issues within the academic publishing domain/s. This review underscores the more subtle, yet equally significant, issue of professional misconduct in the editorial realm of scientific journals.

## Background

Bullying behaviors may be defined as recurring efforts to undermine, unsettle, or evoke fear in a targeted individual or group (Scott et al., 2008). Traditionally, workplace bullying often gravitates toward the well-documented issues of sexual harassment, overt bullying, and acts of discrimination (Cleary et al., 2010; Jagsi et al., 2023). These forms of mistreatment have long been at the forefront of discussions surrounding workplace dynamics and employee wellbeing. Numerous studies and legislative measures have emerged to address and combat these deeply ingrained problems, highlighting the importance of creating inclusive and respectful work environments (Averbuch et al., 2021; Hay, 2021; Moss et al., 2022; Iyer et al., 2023). However, the landscape of professional bullying is far more complex and pervasive than its traditional manifestations. Academic bullying and misconduct have infiltrated the seemingly impartial realm of scientific and scholarly publishing, raising concerns about the very foundations of knowledge dissemination and academic discourse (Smith, 2003; Godlee, 2004). In other words, bullying, a pervasive issue in various professional settings extends beyond traditional workplace bullying; and even involves instances where editors misuse their positions of authority, exhibit hostile behavior, or engage in questionable practices that compromise the integrity of the editorial process (Teixeira and da Costa, 2010).

The role of Editors particularly the Editor-in-Chief/s in scientific journals is pivotal as they are entrusted with safeguarding the rigor and credibility of published research. In other words, the role of Editor/s is vital in maintaining the integrity and credibility of academic discourse. Nevertheless, as with any position of power, there is a potential for misuse leading to a growing concern in the academic community (Godlee, 2004; Faggion, 2021). Such conduct can manifest in various ways, including biased decision-making, suppression of dissenting voices, and the exploitation of power dynamics in the peer review process. In the domain of scientific publishing, considerable attention has been devoted to authorial misconduct, encompassing phenomena like “honorary and/or ghost authorships,” “plagiarism”, “self-citations,” and strategies for upholding publishing ethics (Baskin and Gross, 2011; Al Lamki, 2013; Helgesson and Eriksson, 2015); nevertheless, it is imperative to acknowledge that professional misconduct can also manifest from the editorial perspective (Barbour et al., 2016). According to Zapf et al. (2003), public humiliation, such as belittling comments, is a form of bullying behavior that some editors may display. Moreover, as per Einarsen et al. (2009), publicly berating individuals such as authors of manuscripts submitted to journals in front of colleagues and/or using derogatory language against individuals including authors submitting manuscripts to a journal are clear signs of bullying behavior by journal editors. In this context, ensuring the quality, transparency, and integrity of this process is of paramount importance in scientific publishing, which serves as the cornerstone of academic knowledge dissemination, serving as the gateway to sharing innovative research findings with the global scientific community. Actions, such as those referenced above are indicative of an effort to intimidate authors, a behavior that can significantly hinder the open exchange of ideas and erode the fundamental tenets of scholarly exploration. The World Medical Association (WMA), the (ICMJE), and the Committee on Publication Ethics (COPE) have collectively outlined ethical responsibilities for editors in the publication and dissemination of research findings (International Committee of Medical Journal Editors, 1997; Cockcroft, 2000; Wager, 2012; World Medical Association, 2013). Likewise, the COPE guidelines include a section addressing the handling of misconduct, primarily intended for editors; however, authors should also familiarize themselves with the procedures that editors will follow when addressing suspected misconduct, which involve thorough investigation and resolution of such issues. The present study seeks to contribute to the scientific understanding of editorial bullying within academic journals, exploring the complex dynamics through which journal editors may exhibit bullying behavior; however, it was demanding to cite indexed references for each variable as scholarly articles often focus on broader issues rather than individual cases.

The present mini-review seeks to shed light on these issues, highlighting their gravity and the urgency to address them within the academic publishing landscape.

## What is editor bullying and misconduct?

Editor bullying refers to the abuse and/or misuse of power and influence in positions of authority within academic publishing. It involves behaviors that undermine the scientific process, compromise the integrity of research, and harm the careers and wellbeing of individuals. In addition, the dissemination of submission-related information including details such as manuscript number, title, and editorial decision to colleagues and peers without obtaining prior consent from the authors constitutes a breach of editorial ethics and is considered a form of editorial misconduct (Barbour et al., 2016). In a longitudinal analysis, Petersen AM (Petersen, 2019) investigated the editorial activity of ~7,000 editors affiliated with a well-known journal (denoted as PO) during the 10-year period spanning 2006–2015. The results revealed a striking disparity in power distribution among editors of this reputed journal, with the top 10 editors overseeing a substantial 3,366 articles, constituting 2.4% of the total 141,986 articles scrutinized in this study (Petersen, 2019). Moreover, Petersen (2019) reported that articles handled by extremely active editors of PO had significantly faster acceptance rates, had higher rates of citations to the Editors' research, and lower citation impact relative to the other studies published in this journal. These results provided corresponding lines of evidence supporting an underlying self-citation strategy thereby suggesting either a lack of interest or scientific misconduct on the part of the Editors of PO (Petersen, 2019). This conspicuous inequality raises concerns about inadvertent incentives that may potentially foster biased decision-making at the editorial level, possibly fueling unethical conduct. Results by Petersen (2019) imply that editors could become less discerning in evaluating article quality and may be susceptible to imbalances driven by power. Furthermore, this study (Petersen, 2019) suggested that these effects become more pronounced as editors accumulate experience, in alignment with behavioral research on the evolution of misconduct and susceptibility to temptation in power-driven environments.

## Potential factors associated with editor bullying/misconduct

### Breach of confidentiality

Authors have a legitimate expectation of confidentiality during the peer review process. Unauthorized disclosure of manuscript-related information violates this expectation and can deter authors from submitting their work to that journal or even engaging with the peer review system altogether (Shahan et al., 2006).

### Personal criticism of author/s

In the realm of academia and scholarly discourse, it is essential to maintain a high standard of professionalism and ethical conduct. One fundamental principle that must be upheld is the notion that personal criticism of the author/s is inappropriate. While robust critique and constructive feedback are essential components of the peer review process, they should always focus on the content, methodology, and presentation of the research, rather than targeting the author personally and/or the institution/s of author/s. Personal criticism shifts the focus away from the research and into the monarchy of personal attacks. According to Prasad and Ioannidis (Wallace and Siersema, 2015) “reputational tarnishing”, “degree of moralizing”, and “calls to suspend/censor/retract the work or the author” are forms of obsessive criticism. According to an article published in the journal *Science*, “rude paper reviews are pervasive and sometimes harmful” (Wilcox, 2019). This not only detracts from the intellectual rigor of scholarly discourse but also creates an environment where authors may become reluctant to share their work for fear of personal attacks. Preventive and therapeutic approaches in this context may encompass various strategies, such as enhancing awareness, recognizing and mitigating exacerbating elements, imposing constraints on the quantity of content handled by editors, purposefully pairing commissioned editorials, assimilating contentious discussions from unregulated platforms like social media or PubPeer into the pages of scientific journals, upholding dignity, prioritizing evidence-based discourse, and abstaining from making personal statements/comments (Wallace and Siersema, 2015).

### Intimidation

Author intimidation by journal editors may manifest in several ways, each with varying degrees of severity. The following are common instances of intimidation, often stemming from the editorial process:

#### Unjustified delays/rejections

Editors may unduly delay and/or bypass the review process and render decisions based on personal judgment. This can leave authors in limbo, anxious about the fate of their work and future career prospects (Powell, 2016). In March 2012, Stephen Royle, a cell biologist at the University of Warwick, United Kingdom initiated a personal mission for publication (Powell, 2016). His recent research addressed a contentious question concerning how cells detect the alignment of chromosomes before division. Initially submitted *to Nature Cell Biology*, a well-reputed journal in his field, the manuscript faced rejection without undergoing a review (Powell, 2016). Pending several rejections and resubmissions, Royle's manuscript was accepted for publication pending a delay of 317 days with an additional delay of 53 days until online publication (Powell, 2016). Royle expressed that the multiple rejections had a demoralizing impact, particularly on his student, who needed the study published for graduation (Powell, 2016). Royle further stated that the average time from first submission to publication mirrored that of a human gestation period, ~9 months (Powell, 2016).

#### Harsh or unprofessional feedback

Maintaining a respectful tone in peer reviews is crucial, irrespective of the overall quality of the manuscript. Authors invest significant effort in conducting their studies, preparing, and submitting their papers, often presenting their best work for evaluation. Therefore, it is imperative to maintain a collegial and respectful demeanor, acknowledging and appreciating these dedicated efforts. Editors may provide feedback in a condescending, disrespectful, or unconstructive manner (Zazgyva et al., 2017). This not only undermines the author's confidence but also detracts from the quality of the manuscript revision. If an editor finds fault with the scientific quality of a manuscript, it is imperative to maintain professionalism and objectivity in the critique, refraining from making statements that pertain to the author's representation of their institution based on the perceived inadequacies within the manuscript. Certain journals provide their assigned editors with the discretion to refrain from including unprofessional or discourteous comments when communicating with authors (Mavrogenis et al., 2020). This stands in contrast to other journals with policies that prohibit editors from redacting any content, obliging them to present reviewers' comments to authors in their entirety. The responsibility for overseeing reviewer comments falls on journal editors. In instances where researchers encounter a rude peer review, it is advised not to be disheartened but instead to respond to the editor. Authors can express that they have received a review that they find inaccurate or impolite, and request to be reassigned to another reviewer for a more objective evaluation of their manuscript. However, in cases where an editor-in-chief (the most powerful authority on the journal's platform) may opt to reject a manuscript without conducting a peer review and provides the authors with harsh feedback in an unprofessional manner, whom should the authors contact for communication? It is suggested that the assessment of scientific merit should remain focused on the content and methodology of the research rather than making implications and/or generalizations about the author's experience in the field of study, age, gender and/or institutional affiliation. According to Nobarany and Booth (2015), inexperienced researchers exhibited a higher frequency of unreserved criticism compared to their experienced counterparts. Additionally, reviewers tended to employ positive politeness strategies, such as compliments, more frequently when engaging with less experienced authors (Nobarany and Booth, 2015).

### Editors as authors in their own journals—Navigating ethics and integrity

The phenomenon of editors publishing multiple articles within their own journal/s warrants careful consideration from an ethical standpoint. In a systematic review, Helgesson et al. (2022) assessed studies on the prevalence of editors publishing in their own journals and performed a regularizing ethical evaluation of this practice. In this systematic review (Helgesson et al., 2022), 15 studies were included. The results showed that self-publishing varied significantly across fields, journals, and editors, encompassing those who refrained from publishing in their own journal to others who extensively contributed to their own journal (Helgesson et al., 2022). The authors also noted that numerous studies incorporated into their systematic review exhibited methodological inaccuracies (Helgesson et al., 2022). However, findings of this systematic review (Helgesson et al., 2022) indicated instances where self-publication rates were notably elevated. Helgesson et al. (2022) proposed that editors-in-chief and associate editors, vested with substantial influence in their respective journals, abstain from publishing research articles within the journals they oversee. Helgesson et al. (2022) further proposed that journals should establish stringent and transparent protocols regarding the handling of manuscripts submitted by members of the editorial board. Authors of the present mini-review applaud the study by Helgesson et al. (2022) as such practices may raise concerns about the potential compromise of the peer review process, as editorial oversight may be perceived as influencing the objectivity and rigor of the evaluation. Moreover, repeated publication of studies authored by journal editors within their own publication domain may create an appearance of impropriety, casting doubt on the impartiality and fairness of the editorial decision-making process. From a scholarly perspective, this may undermine the principle of merit-based publication, where contributions are assessed solely on their academic and scientific merits. Furthermore, the act of publishing multiple articles within one's own journal may contribute to the distortion of citation metrics and impact factors, influencing the perceived significance and impact of the journal within the academic community. Ethical guidelines within the scientific community emphasize the importance of transparency, fairness, and avoidance of conflicts of interest. Therefore, editors engaging in the practice of publishing multiple articles within their own journal should be vigilant in adhering to rigorous editorial standards and transparent disclosure of potential conflicts, thereby upholding the integrity and credibility of the scientific publishing process.

## Recommendation/anonymous surveys

Typically, publishers extend invitations to authors to partake in surveys assessing their experiences with the publication process. However, authors of the present mini-review propose that journals should also proactively invite authors to participate in anonymous surveys specifically focused on the submission, review process and communication with the handling editor and editor-in-chief. The implementation of such anonymous surveys holds the potential to uncover and address any potential conflicts of interest and/or controversies that may arise during manuscript submission, interactive review, and decision phases, which might otherwise remain undisclosed in the final published article. Engaging in such initiatives can also serve as a valuable educational tool for editors, reviewers, and prospective authors submitting future contributions.

## Conclusion

The pervasive issue of bullying and misconduct in professional settings and their negative impact on academic publishing cannot be overlooked. The implementation of routine mandatory educational training for authors, reviewers, and editors may serve as a proactive measure that can effectively mitigate the risk of bullying within the realm of scientific publishing.

## Author contributions

FJ: Conceptualization, Methodology, Writing—original draft, Writing—review & editing. DM: Methodology, Writing—original draft, Writing—review & editing. PR: Supervision, Writing—original draft, Writing—review & editing.
